# Exclusive expression of the Rab11 effector SH3TC2 in Schwann cells links integrin-α_6_ and myelin maintenance to Charcot-Marie-Tooth disease type 4C

**DOI:** 10.1016/j.bbadis.2016.04.003

**Published:** 2016-07

**Authors:** Sauparnika Vijay, Meagan Chiu, Joel B. Dacks, Rhys C. Roberts

**Affiliations:** a Cambridge Institute for Medical Research, Department of Clinical Neurosciences, University of Cambridge, Cambridge Biomedical Campus, Cambridge CB2 0XY, UK; b Department of Cell Biology, University of Alberta, 5-31 Medical Science Building, Edmonton, Alberta, Canada

**Keywords:** Charcot-Marie-Tooth disease, Schwann cells, Peripheral neuropathy

## Abstract

Charcot-Marie-Tooth disease type 4C (CMT4C) is one of the commonest autosomal recessive inherited peripheral neuropathies and is associated with mutations in the Rab11 effector, SH3TC2. Disruption of the SH3TC2–Rab11 interaction is the molecular abnormality underlying this disease. However, why *SH3TC2* mutations cause an isolated demyelinating neuropathy remains unanswered. Here we show that SH3TC2 is an exclusive Schwann cell protein expressed late in myelination and is downregulated following denervation suggesting a functional role in myelin sheath maintenance. We support our data with an evolutionary cell biological analysis showing that the *SH3TC2* gene, and its paralogue *SH3TC1*, are derived from an ancestral homologue, the duplication of which occurred in the common ancestor of jawed vertebrates, coincident with the appearance of Schwann cells and peripheral axon myelination. Furthermore, we report that SH3TC2 associates with integrin-α_6_, suggesting that aberrant Rab11-dependent endocytic trafficking of this critical laminin receptor in myelinated Schwann cells is connected to the demyelination seen in affected nerves. Our study therefore highlights the inherent evolutionary link between SH3TC2 and peripheral nerve myelination, pointing also towards a molecular mechanism underlying the specific demyelinating neuropathy that characterizes CMT4C.

## Highlights

• Mutations in SH3TC2 cause the demyelinating peripheral neuropathy, CMT type 4C.
• The SH3TC2 protein is exclusively expressed in myelinating Schwann cells.
• SH3TC2 is expressed late in myelination suggesting a role in myelin maintenance.
• The *SH3TC2* gene emerged concurrently with the appearance of Schwann cells.
• SH3TC2 associates with integrin-α_6_, which is expressed in myelinating Schwann cells.

## 1 Introduction

Progressive degeneration of peripheral nerves is the pathological hallmark of the Charcot-Marie-Tooth diseases (CMT), the most common inherited neuromuscular disorder. Clinically, CMT is characterized by muscle wasting and weakness, sensory loss and limb deformities [1]. Over 80 genes have now been shown to be associated with CMT [2], highlighting key factors that are essential for peripheral nerve development and function.

Peripheral nerves are composed of axons and Schwann cells. Indeed, in keeping with this anatomical dichotomy, CMT can also be classified into ‘demyelinating’ or ‘axonal’ forms, reflecting the main sites of pathology as the Schwann cell or axon, respectively [3]. Schwann cells play vital supportive roles including the formation of myelin sheaths required for the efficient conduction of action potentials along axons. Schwann cell myelination of axons is highly regulated and is also characterized by the sequential segregation of ion-channels and scaffolding proteins to form specialized domains along the peripheral nerve such as the node of Ranvier, juxtaparanode and the paranodal region. Disruption of these regions can lead to peripheral nerve dysfunction and a peripheral neuropathy [4].

Myelination of peripheral nerve axons by Schwann cells is dependent on the expression of specific isoforms of the integrin family of laminin receptors [5]. These integral membrane proteins are targeted to the plasma membrane where they function as adhesion molecules by attaching to the extracellular basal lamina. Integrin-α_6_ in complex with integrin-β_1_ is expressed in Schwann cells early in the process of myelination, followed later as a complex with integrin-β_4_, which is critical in maintaining the structural stability of the mature myelin sheath [6], [7]. Mice lacking the α_6_/β_4_ receptor in Schwann cells display normal myelination initially, but develop myelin instability with time. Integrins are known to be trafficked in the endocytic and secretory pathways, and also regulate downstream intracellular signaling [8]. Specifically, integrin-α_6_/β_4_ has long been known to undergo endocytic recycling [9], with Rab11 also shown to regulate its cell surface expression [10].

Rab11 has also previously been linked with CMT type 4C, an autosomal recessive demyelinating form of CMT characterized by an early-onset neuropathy, with scoliosis as a prominent clinical feature [11]. The disease is associated with mutations in *SH3TC2*, which encodes a predicted 144 kDa protein containing two N-terminal SH3 domains and at least six C-terminal tetratricopeptide repeat motifs (TPR). Both types of domain are thought to mediate protein–protein interactions. Interestingly, mutations in *SH3TC2* were the first mutations to be described following the application of next generation sequencing technologies to a previously undiagnosed family with a recessive form of CMT [12]. Previously, we and others have shown that epitope-tagged SH3TC2 targets to intracellular membranes [13], [14], [15]. Specifically, we reported that SH3TC2 tagged with green fluorescent protein (GFP) targets to the endocytic recycling compartment and is a Rab11 effector that affects the endocytic recycling of transferrin receptors (TfR) when expressed in HeLa cells. Moreover, all reported CMT4C-associated pathogenic mutations in SH3TC2 led to loss of intracellular targeting, loss of Rab11 binding, and loss of function on the endocytic recycling pathway. This led us to propose that disruption of the SH3TC2–Rab11 interaction is the fundamental molecular abnormality that underlies CMT4C [13]. Nevertheless, it remains unclear why mutations in *SH3TC2* lead exclusively to a demyelinating peripheral neuropathy.

No study has yet to definitively describe where and when the SH3TC2 protein is expressed in humans or mice. Establishing the expression pattern of the endogenous protein is therefore vital both to understand why mutations in *SH3TC2* lead specifically to a demyelinating peripheral neuropathy and also to devise potential therapeutic strategies for this and other subtypes of CMT. In addition, which membrane cargo proteins require the presence of SH3TC2 remains unclear.

We now report that SH3TC2 is found exclusively in myelinating Schwann cells, and is expressed late during myelination. Furthermore, SH3TC2 is rapidly downregulated following denervation. To better understand the significance of this dynamic expression pattern, we also conducted a comprehensive evolutionary study of the *SH3TC2* gene and its uncharacterized paralogue, *SH3TC1*, which supports the hypothesis that SH3TC2 is a key protein whose evolutionary appearance coincides with that of Schwann cells and myelination of peripheral axons. Furthermore, we report that SH3TC2 associates with the laminin receptor, integrin-α_6_, providing a mechanistic link between SH3TC2 and the structural maintenance of the myelin sheath. Given that SH3TC2 is expressed late in the process of myelination, our work also identifies a potential temporal window for future therapeutic intervention applicable to patients diagnosed with CMT4C.

## 2 Materials and methods

### 2.1 Reagents and cell culture

**Fig. 1:**
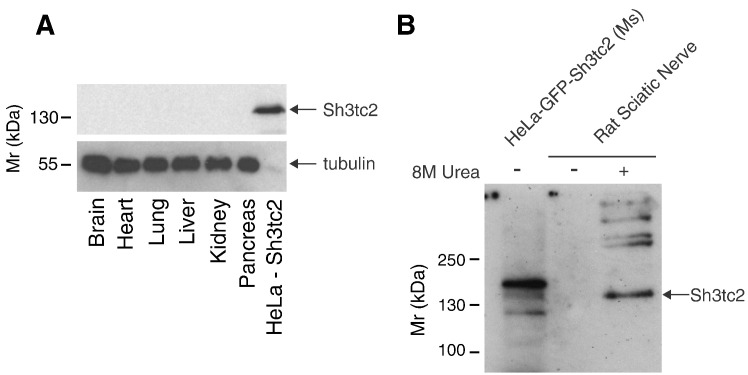
Sh3tc2 is expressed only in sciatic nerve. A. Western blot of a panel of rat tissue lysates to determine endogenous tissue expression of Sh3tc2. A cell lysate from HeLa cells transiently expressing GFP–Sh3tc2 was used as a positive control and tubulin used to compare protein loading between tissues. Note that GFP–Sh3tc2 transiently expressed in HeLa cells is easily detected despite the relatively low concentration of tubulin compared to the rat tissues. B. Western blot of rat sciatic nerve lysate to determine endogenous expression of Sh3tc2. Rat sciatic nerve lysate was prepared as in A by homogenization in lysis buffer containing 1% Ipegal, 0.5% deoxycholate and 0.1% SDS before centrifugation. The remaining insoluble pellet was dissolved in 8 M Urea. Endogenous Sh3tc2 was found only in the detergent-insoluble pellet (marked ‘+’) with no Sh3tc2 detected in the absence of 8 M Urea (marked ‘−’). A cell lysate from HeLa cells transiently expressing GFP–Sh3tc2 was used as a positive control.

**Fig. 2:**
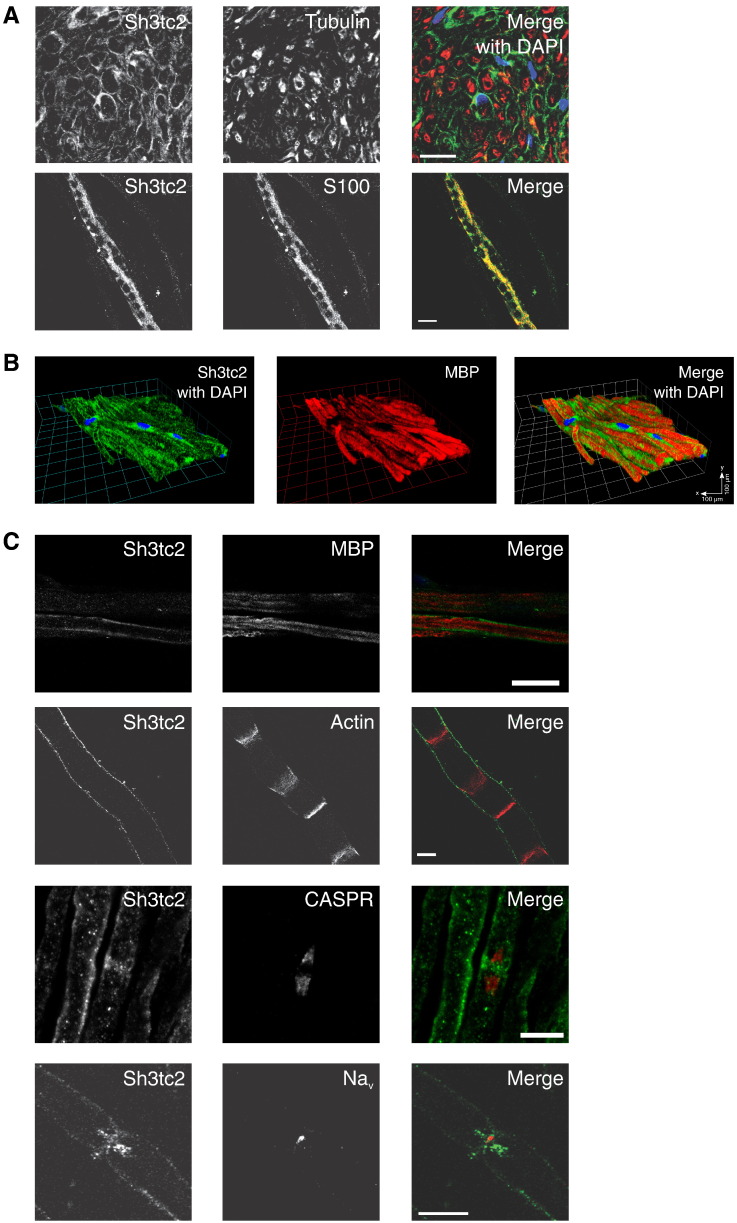
Sh3tc2 is expressed in Schwann cells. A. Immunofluorescence studies of teased rat sciatic nerves visualized by confocal microscopy. The top panel shows a transverse section of sciatic nerve with Sh3tc2 (green) displaying crescent-shaped staining distinct from axonal tubulin (red), confirming that Sh3tc2 is expressed in Schwann cells. A merged image on the right includes nuclei stained with 4′-6-diamidino-2-phenylindole (DAPI). The lower panel shows a 3 dimensional render of confocal z stacks of a rat sciatic nerve teased fiber stained for Sh3tc2 (green) and the Schwann cell cytoplasmic marker, S100 (red) with a merged image seen on the right (Volocity 6.2, PerkinElmer). The resulting images are reminiscent of Cajal bands, which are Schwann cell cytoplasmic channels that run longitudinally along axons and are critical in maintaining the integrity of the myelin sheath. Scale bars denote 20 μm. B. Three dimensional render of confocal z stacks of teased rat sciatic nerve fibers stained for Sh3tc2 (green), MBP (myelin basic protein, red) and DAPI (blue). The figure shows that Sh3tc2 is found in Schwann cell cytoplasm abutting compact myelin, as indicated by MBP. C. Colocalization immunofluorescence studies of rat sciatic nerve teased fibers by confocal microscopy. Sh3tc2 is shown in the left panel and endogenous marker proteins are shown in the middle panel. Merged images containing Sh3tc2 (green) and the indicated endogenous protein (red) are shown on the right. The figure shows that Sh3tc2 is found in Schwann cell cytoplasm abutting compact myelin, as indicated by MBP, but is not found in Schmidt–Lanterman incisures (Actin), the paranode (CASPR) or the node of Ranvier (Na_v_). Scale bars denote 20 μm apart from lowest panels showing Na_v_ staining where scale bar denotes 10 μm.

**Fig. 3:**
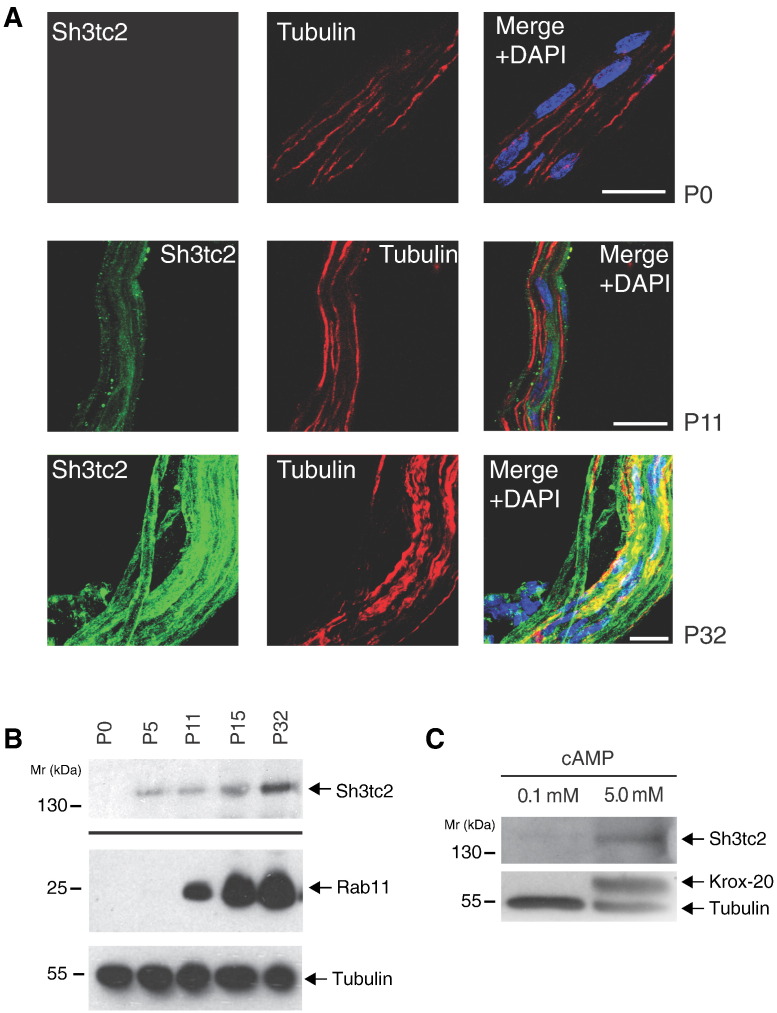
Sh3tc2 is expressed late in the process of myelination. A. Immunofluorescence studies of Sh3tc2 expression in teased rat sciatic nerves at 0, 11 and 32 days after birth as visualized by confocal microscopy. Merged images are shown on the right with nuclei stained with 4′-6-diamidino-2-phenylindole (DAPI). Scale bars denote 20 μm. B. Western blot of sciatic nerve lysates taken from rats at 0, 5, 11, 15 and 32 days after birth. The top panel represents the detergent-insoluble fraction from processed sciatic nerves following solubilization in 8 M Urea. Protein loading was determined and normalized by Coomassie staining following SDS-PAGE (data not shown). The lower panels show the corresponding expression of the Sh3tc2-interacting protein, Rab11 and tubulin, which are both found in the detergent-soluble lysate. C. Western blot of primary rat Schwann cells incubated with 5 mM cAMP to chemically activate the myelination transcription program. cAMP induces the expression of Sh3tc2 as well as Krox-20, the key transcription factor controlling the myelination program. The lower panel was first immunoblotted for tubulin and then re-probed with an antibody towards Krox-20.

**Fig. 4:**
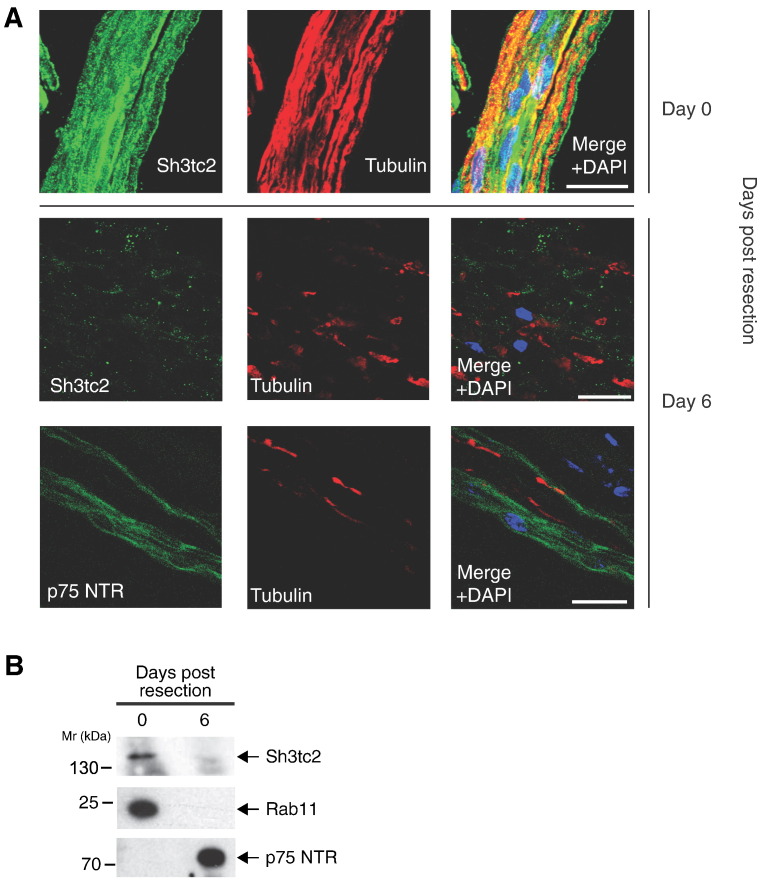
Sh3tc2 is downregulated following axon injury. A. Immunofluorescence studies of teased rat sciatic nerves immediately following resection and after 6 days incubation in DMEM media as visualized by confocal microscopy. Merged images on the right show Sh3tc2 or p75 NTR (green), tubulin (red) and nuclei stained with 4′-6-diamidino-2-phenylindole (DAPI). Scale bars denote 20 μm. B. Western blot showing that the expression of Sh3tc2 in rat sciatic nerve is downregulated following resection and incubation in DMEM media for 6 days. In contrast, as the nerve degenerates, Schwann cells upregulate p75^NTR^.

Antibodies used during this study include: rabbit anti-SH3TC2 (Abcam) (Fig. 1, Fig. 2, Fig. 3, Fig. 4), mouse anti-S100 (Abcam), mouse anti-Caspr (Abcam), mouse anti-myelin basic protein (Abcam), mouse anti-α-tubulin (AA4.3, DSHB [16]), mouse anti-beta III tubulin (Abcam), goat anti-EF2 (Santa Cruz), rabbit anti-GAPDH (Cell Signaling), mouse anti-sodium channel (Sigma), rabbit anti-p75 NGF receptor (Abcam), Rabbit anti-Krox-20 (Covance), mouse anti-GFP (Roche), mouse anti-myc (Cell Signaling), rat anti-ITGA6 (Biolegend), mouse anti-LAMP1 (H4A3, August, J.T./Hildreth, J.E.K., DSHB), Alexa Fluor 568 phalloidin (Invitrogen), Alexa Fluor 488- and Alexa Fluor 568-conjugated goat anti-rabbit, anti-mouse, anti-rat and Alexa Fluor 488-conjugated donkey anti-rabbit and Alexa Fluor 555-conjugated donkey anti-goat secondary antibodies (Invitrogen). A rabbit polyclonal antibody (2058) was also generated against residues 350 to 500 of human SH3TC2 (Eurogentec, Belgium) and affinity-purified (Fig. 7). HRP-conjugated goat anti-rabbit, goat anti-mouse, goat anti-rat and donkey anti-goat antibodies were used as secondary antibodies for western blotting (Sigma-Aldrich). Protein bands separated by SDS-PAGE and transferred to nitrocellulose membranes were detected using WesternBright ECL Western blotting detection kit (Advansta, CA, USA). Supersignal West Femto (Thermo Fisher) was used for detection of integrin-α_6_ expression in cell lysates.

Full length mouse Sh3tc2 cDNA was obtained from the RIKEN bioresource center (Clone ID F420014A15). The full length mouse Sh3tc2 clone (corresponding to NCBI Reference Sequence: NM_172628.2) was generated by addition of the missing 191 3′-base pairs using a synthesized gBlock (Integrated DNA Technologies, Iowa, USA) and restriction free cloning [17], [18] into pEGFP-C1 vector (Clontech). YFP-SH3TC1 was purchased from Source Bioscience (Cambridge, UK). DNA constructs were sequenced and validated by Source Bioscience (Cambridge, UK).

HeLaM cells were grown at 37 °C in RPMI (Sigma-Aldrich) containing 10% FCS and 2 mM l-glutamine, 100 U/ml penicillin and 100 μg/ml streptomycin in a 5% CO_2_ humidified atmosphere. HeLa cells were transfected using polyethylenimine (Polysciences, PA, USA). RPE cells were cultured at 37 °C in DMEM:Ham's F-12 (50:50) (Sigma-Aldrich), 10% FCS, 2 mM l-glutamine, 100 U/ml penicillin and 100 μg/ml streptomycin in a 5% CO_2_ humidified atmosphere.

Schwann cells (SCs) were cultured according to a modified protocol from Kaewkhaw et al. [19]. Sciatic nerves were dissected from adult rats and SCs were allowed to dedifferentiate in DMEM supplemented with 10% FCS and 100 U/ml penicillin and 100 μg/ml streptomycin for five days. Epineurium and perineurium were removed and the tissue was digested in dispase and collagenase. The cells were seeded on PDL/laminin coated dishes in DMEM/D-valine (PAA laboratories) supplemented with 10% FCS, 1% penicillin and streptomycin, bovine pituitary extract, forskolin, 1% N2 supplement and 10 ng/ml neuregulin. The media was supplemented with 5 mM cyclic AMP to induce chemical myelination.

### 2.2 Immunohistochemistry of Sciatic Nerves

Sciatic nerves were dissected from rats and immersion-fixed in 4% paraformaldehyde (PFA) (w/v) for 30 min. The nerves were stored in PBS overnight. Epineurium and perineurium were removed and individual fibers were teased on charged glass slides (Superfrost plus, Fisher scientific), air dried and stored at − 80 °C. The teased fibers were then treated with ice-cold methanol for 10 min and rehydrated before immunostaining. For cross-sections, fixed nerves were cryoprotected in 30% sucrose (w/v) in PBS and embedded in optimal cutting temperature compound. 10 μm sections were cut using a cryostat; air dried and stored at − 80 °C. Tissues/cells were blocked for 30 min in PBS with 10% fetal calf serum, 10 mM glycine and 3 mM sodium azide at room temperature. The tissues were incubated with primary antibodies overnight at 4 °C and secondary antibodies for 1 h at RT. Wide-field fluorescent images were obtained using a Zeiss Axioplan Fluorescent Microscope and confocal images were obtained using Zeiss LSM510 META and Zeiss LSM880 confocal microscopes (Carl Zeiss). Data obtained from the confocal microscopes were analyzed using Zeiss LSM and ZEN software (Carl Zeiss), and Volocity (PerkinElmer).

### 2.3 Protein extraction for western blotting

Tissues were homogenized in lysis buffer (50 mM Tris–HCl pH 8, 150 mM NaCl, 0.5 mM EDTA, 1% Igepal, 0.5% deoxycholate, 0.1% SDS and Complete protease inhibitors (Roche, UK)) at 4 °C. The lysates were then briefly sonicated at 4 °C before centrifugation for 30 min at 21,000 ×* g* and the resulting supernatant boiled in SDS-sample buffer (200 mM Tris–HCl pH 6.8, 30% glycerol, 2.4 M 2-mercaptoethanol, 0.1% SDS, 0.25% bromophenol blue (w/v)). For sciatic nerves, a visible detergent-insoluble pellet was obtained in addition to a detergent-soluble fraction and the pellet was further processed by dissolving in 8 M Urea before boiling in sample buffer. Proteins were separated by SDS-PAGE and transferred to nitrocellulose membranes by wet or semi-dry electrotransfer. Equal loading of protein per lane was achieved following relative protein concentration determination of Coomassie-stained protein lysates using a LI-COR Odyssey Quantitative Scanning System (LI-COR Biosciences) [20].

### 2.4 Comparative genomics

Complete predicted proteome data derived from genome databases for the following taxa were downloaded from the National Center for Biotechnology Information (NCBI) or the joint genome institute (JGI) including: *Homo sapiens*, *Rattus norvegicus*, *Mus musculus*, *Ornithorhynchus anatinus*, *Anolis carolinensis*, *Gallus gallus*, *Meleagris gallopavo*, *Taeniopygia guttata*, *Xenopus tropicalis*, *Danio rerio*, *Branchiostoma floridae*, *Ciona intestinalis*, *Drosophila melanogaster*, *Caenorhabditis elegans*, *Trichoplax adhaerens*, *Monosiga brevicollis*, *Salpingoeca* sp., *Capsaspora owczarzaki*, *Sphaeroforma arctica*, *Rozella allomycis*, *Encephalitozoon cuniculi*, *Piromyces* sp., *Batrachochytrium dendrobatidis*, *Catenaria anguillulae*, *Coemansia reversa*, *Conidiobolus coronatus*, *Rhizophagus irregularis*, *Schizosaccharomyces pombe*, *Saccharomyces cerevisiae*, *Cryptococcus neoformans*, *Nematostella vectensis* and *Callorhinchus milii*. Additionally, data were downloaded from ensembl (*Latimeria chalumnae*, *Petromyzon marinus*, *Amphimedon queenslandica*, and *Takifugu rubripes*) and the Broad institute (*Thecamonas trahens*, *Fonticula alba*, *Nosema ceranae*, *Mortierella verticillata*, *Rhizopus delemar*, *Neurospora crassa*, and *Ustilago maydis*).

The *H. sapiens* sequences of SH3TC1 (NP_061859.3) and SH3TC2 (NP_078853.2) were used as starting queries. Initial searches were undertaken into the respective organismal databases with these queries using the basic local alignment search tool (BLAST) performed locally. The resulting potential homologues were listed from most similar to least similar and ranked by E-value. Results with an E-value equal to or greater than 0.05 were regarded as a negative hit and not subjected to further analysis. For increased sensitivity, the search process was repeated using HMMer (http://www.hmmer.org), again using the above human sequences as starting queries and keeping a cut-off e-value of 0.05 as a candidate for a putative positive hit. In all cases, putative homologues were subjected to reciprocal BLAST analysis and used as queries into the downloaded *H. sapiens* predicted proteome. Proteins were deemed as legitimate orthologues if they retrieved the initial human query sequences or a named version thereof with an e-value of 0.05 or lower.

In order to further search for *SH3TC* genes in the crucial sampling point of the *C. milii* genome, searches were undertaken of the nucleotide data available from this taxon, downloaded from NCBI. BLASTx searches using the above *H. sapiens* proteins as queries were undertaken, yielding hits on nucleotide scaffolds to open reading frames (ORFs) corresponding to an unknown *C. milii* protein. The conceptual protein sequence was obtained by manual in silico removal of introns, yielding the longest possible ORF that showed homology to the *SH3TC* genes across its entire length.

### 2.5 Phylogenetic analysis

All sequences identified as SH3TC homologues (listed by accession number, figure designation and abbreviation in Table S1) were aligned using MUSCLE [21]. Multiple sequence alignments were assessed by eye using Mesquite v2.75 and only regions of unambiguous homology were retained for phylogenetic analysis. ProtTest v.3.3 [22], [23] was used to assess the most appropriate model of sequence evolution. Phylogenetic trees generated using PhyloBayes v1.5a [24] and RAxML v8.0.24 [25], bootstrapped with 100 pseudoreplicates. RAxML consensus trees were produced using the consense program from the Phylip package v3.695 [26]. Phylogenetic trees were viewed using FigTree v1.4, and exported to produce final figures in Adobe Illustrator CS4. Initial analyses of a 28 taxon, 1200 position matrix, run using a model of PROTGAMMAIJTTF for RAxML and LG for PhyloBayes, clearly showed that the duplication of SH3TC into SH3TC1 and SH3TC2 occurred prior to the speciation of jawed fish, with the three identified *C. milii* sequences robustly within the SH3TC1 clade (see Supplementary Fig. 6). Due to the branch length of the manually predicted *C. milii* SCH3TC1-c sequence, the analysis was repeated (run under a PROTGAMMAJTTF model for RAxML and an LG model for PhyloBayes) with the sequence excluded (Fig. 6B), yielding a dataset of 27 taxa and 1125 positions. All alignments are available upon request.

### 2.6 BioID

RPE cells stably expressing either BirA or BirA-SH3TC2 were generated using the pLXIN retroviral system (Clontech) as previously described [27] and incubated with 50μM biotin (Sigma-Aldrich) for 24 h at 37 °C. Cells were then scraped into ice-cold PBS, pelleted and washed twice with PBS. Cell lysis was performed by resuspension in lysis buffer (50 mM Tris–HCl pH 7.4, 150 mM NaCl, 1% Ipegal, 0.5% sodium deoxycholate, 1 mM EDTA, 0.1% SDS and Complete protease inhibitor (Roche, UK)) followed by gently passing the lysate through a 25G needle. The lysates were then incubated on ice for 10 min before sonication and centrifugation at 20,000 ×* g* for 15 min. Following an overnight incubation of the lysate with streptavidin beads (Thermo Scientific, USA) at 4 °C, the beads were washed with lysis buffer, TBS (50 mM Tris–HCl pH 7.4, 150 mM NaCl) and finally with 50 mM ammonium bicarbonate. 50 mM ammonium bicarbonate supplemented with 10 mM DTT was then added to the beads and heated for 30 min at 56 °C followed by the addition of 50 mM iodoacetamide and further incubation at room temperature in the dark. The beads were washed twice with 50 mM ammonium bicarbonate before incubation with 1 μg of trypsin (Roche, UK) in 50 mM ammonium bicarbonate overnight at 37 °C. A further 1 μg of trypsin in 0.01% trifluoroacetic acid (TFA) was added to the beads and incubated at 37 °C for 2 h before centrifugation at 300 × g for 5 min. The supernatant and two further washes containing the tryptic peptides were transferred to a fresh tube and spun at 20,000 ×* g* for 10 min to remove insoluble material. The peptides were then transferred to a fresh tube and dried in a vacuum centrifuge. The dried samples were re-suspended in 20 μl MS solvent (3 % MeCN, 0.1 % TFA) for analysis by LC–MSMS using an Orbitrap XL (Thermo) coupled to a nanoAcquity UHPLC (Waters). Peptides were eluted by a gradient rising from 8 to 25 % MeCN by 75 min and 40 % MeCN by 90 min. MS spectra were acquired in the Orbitrap between 300 and 2000 m/z, and MS/MS spectra were acquired in the LTQ in a top6 DDA fashion for ions over 1000 counts. Data was processed in MaxQuant 1.5.0.12.

### 2.7 Immunoprecipitation

RPE cells stably expressing either BirA alone or BirA-SH3TC2 were scraped into a small volume of ice-cold lysis buffer (20 mM HEPES pH 7.4, 150 mM NaCl, 1 mM EDTA, 1% Ipegal and Complete protease inhibitors (Roche, UK)) and incubated on ice for 30 min. The lysates were then sheared by passing the solution through a 23G needle before centrifugation at 20,000 ×* g* for 30 min. Lysates were precleared by incubating with protein A Sepharose beads (GE Healthcare, UK) for 1 h at 4 °C with gentle agitation. The precleared lysates were then incubated with anti-SH3TC2 antibody (2058) overnight at 4 °C with gentle agitation. Fresh protein A Sepharose beads were then added and incubated at 4 °C for 1 h. The beads were spun at low speed and washed thrice with lysis buffer and thrice with PBS. Proteins were extracted by the addition of SDS sample buffer to the beads and incubation at 65 °C for 15 min. Extracted proteins were separated by SDS-PAGE before transfer to nitrocellulose membranes for western blotting.

### 2.8 Duolink proximity ligation assay

The Duolink Proximity Ligation Assay (PLA) (Sigma-Aldrich) was performed according to the manufacturer's guidelines. In brief, cells were plated onto glass coverslips before fixation with 4% PFA (w/v) at 37 °C for 10 min. Cells were blocked with blocking solution for 30 min before incubation with primary antibodies overnight at room temperature. Following washing, the cells were incubated with PLA probes for 1 h at 37 °C, washed twice, before incubating with ligase solution for 30 min at 37 °C. A further two washes were performed before signal amplification by incubating with polymerase solution for 100 min at 37 °C in the dark. The cells were then washed before mounting on glass slides and analyzed using a Zeiss LSM880 confocal microscope.

## 3 Results

### 3.1 Sh3tc2 is expressed exclusively in Schwann cells

Having validated and verified a commercially-available anti-SH3TC2 antibody along with an affinity-purified antibody raised in rabbits towards residues 350–500 of human SH3TC2 (Supplementary Fig. 1A, B, C, D), we first asked which tissues contain endogenous Sh3tc2. By probing a panel of lysates prepared from detergent-solubilized rat tissues, including sciatic nerves, no expression of the Sh3tc2 protein was detected as determined by immunoblotting (Fig. 1A and Supplementary Fig. 2A). However, we noted that, in contrast to the other tissues, there remained a visible insoluble pellet following detergent-solubilization of sciatic nerves. Significantly, following solubilization of this pellet from sciatic nerves with 8 M Urea, we were able to detect the Sh3tc2 protein by western blot (Fig. 1B). Furthermore, multiple higher molecular weight bands were also visualized, suggesting possible oligomerization of Sh3tc2. Importantly, direct solubilization of tissues with 8 M Urea did not result in the detection of Sh3tc2 expression (Supplementary Fig. 2C). These data suggest that Sh3tc2 is only expressed in peripheral nerves.

To establish which peripheral nerve cell type expresses Sh3tc2, we used immunohistochemistry to visualize Sh3tc2 expression in teased adult rat sciatic nerve fibers. Fig. 2A shows that Sh3tc2 is found at the periphery of axons, forming a crescent-shaped staining distinct from axonal tubulin when viewed in cross-section. This indicates that Sh3tc2 is expressed in Schwann cells. Confocal microscopic images following co-staining of teased sciatic nerve fibers with reagents labeling specific subcellular compartments of Schwann cells revealed that Sh3tc2 was located adjacent to the compact myelin sheath (MBP) (Fig. 2B, C). Sh3tc2 was not found at Schmidt–Lantermann incisures (Actin), paranodes (CASPR) or at the nodes of Ranvier (voltage-gated sodium channel) (Fig. 2C). A 3D render of confocal Z stacks taken of teased nerve fibers showed that Sh3tc2 colocalized with the Schwann cell cytoplasmic marker, S100, and was present in longitudinal Schwann cell cytoplasmic channels known as Cajal bands (Fig. 2A), structures that are essential for the structural integrity of myelinated peripheral nerves [28]. Sh3tc2 was not found to colocalize with the adhesion molecule, L1, suggesting that Sh3tc2 is not expressed in Remak cells (Supplementary Fig. 3). These data therefore demonstrate that the Sh3tc2 protein is exclusively expressed in Schwann cells of mature peripheral nerves.

### 3.2 Sh3tc2 expression is upregulated with myelination

Histological evidence of hypomyelination in Sh3tc2 knock-out mice cannot be detected until 56 days after birth by light microscopy, although earlier changes have been reported using electron microscopy [29]. These findings suggest that the formation of the myelin sheath does not depend on Sh3tc2 expression. In keeping with this hypothesis, immunohistochemistry on teased nerve fibers from rats revealed no expression of Sh3tc2 at birth, in contrast to the strong expression of Sh3tc2 seen at 32 days after birth (Fig. 3A). Sh3tc2 expression was also only weakly detected by immunoblotting of sciatic nerves taken from mice at 0, 5, 11 and 15 days after birth, compared to the strong expression detected in 32 day old nerves (Fig. 3B). Interestingly, the expression of the Sh3tc2-interacting protein, Rab11, increases in parallel with Sh3tc2, suggesting a regulated program of expression of these proteins as myelination proceeds. These findings support a functional role for Sh3tc2 in mature myelinated peripheral nerves.

We next focused on the expression of endogenous Sh3tc2 in primary rodent Schwann cells, purified using established methods [30]. The resulting primary Schwann cells become detached from axons during the purification process, with consequent reversal of the myelination program due to the loss of the necessary axonal-derived activating signals. We did not detect Sh3tc2 expression in purified Schwann cells at rest. However, consistent with our previous observations, Sh3tc2 was detected following incubation of purified primary Schwann cells with high concentrations of cAMP (known to activate the myelination program) [30], in tandem with expression of the key myelination transcription factor, krox-20 (Fig. 3C). These data suggest that expression of Sh3tc2 is linked to the activation of Schwann cell myelination.

### 3.3 Sh3tc2 is downregulated following denervation

Following nerve injury, both myelinating and unmyelinating Schwann cells detach from damaged axons and activate a highly regulated signaling programme that optimizes eventual nerve repair and re-innervation of target tissues [31]. This process, orchestrated by the transcription factor c-jun, involves significant morphological changes in Schwann cells and the downregulation of proteins involved in myelination [32]. We therefore used an in vitro model of nerve injury comparing protein expression in rat sciatic nerves immediately following extraction (Day 0) with protein extracted after 6 days of in vitro culture, which mirrors the ‘trans-differentiation’ of Schwann cells that occurs with axonal degeneration in vivo (Day 6) [33], [34]. As predicted for a protein involved with myelination, Sh3tc2 was downregulated 6 days after extraction (Fig. 4A, B), in contrast to the known upregulation of p75^NTR^ neurotrophin receptor on the Schwann cell surface following denervation.

### 3.4 The SH3TC2 homologue, SH3TC1, also targets to the recycling endosome

**Fig. 5:**
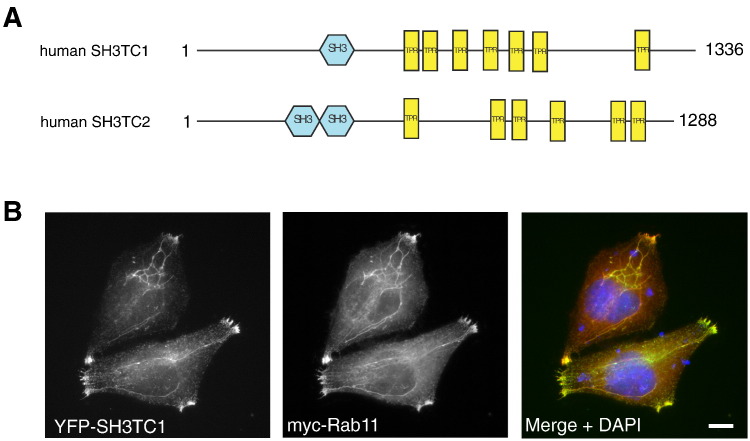
SH3TC1 and SH3TC2 are homologues that target to the endocytic recycling compartment. A. Schematic diagrams showing the predicted domain organization of SH3TC1 and SH3TC2. Domains were predicted using SMART (simple modular architecture research tool — http://smart.embl-heidelberg.de). B. Immunofluorescence studies of HeLa cells transiently expressing YFP-tagged SH3TC1 and myc-tagged Rab11. Merged images on the right show YFP-SH3TC1 (green), myc-Rab11 (red) and nuclei stained with 4′-6-diamidino-2-phenylindole (DAPI). Scale bars denote 10 μm.

The domain architecture of SH3TC2, containing two N-terminal SH3 domains and multiple C-terminal TPR motifs (Fig. 5A), is only shared by one other protein in humans, SH3TC1. *SH3TC1* is an uncharacterized gene found on chromosome 4 and is predicted to encode a 1336 amino acid, 147 kDa protein containing one N-terminal SH3 domain and at least seven C-terminal TPR motifs (Fig. 5A). The human SH3TC1 protein has 37% sequence identity with human SH3TC2 (Supplementary Fig. 4), pointing towards potentially similar functional roles. Moreover, the amino acid residues associated with missense mutations in SH3TC2 leading to CMT4C are all conserved (marked by * in Supplementary Fig. 4). We therefore asked whether SH3TC1 also targets to the endocytic recycling compartment. Indeed, as shown in Fig. 5B, epitope-tagged SH3TC1 targeted to the endocytic recycling compartment when transiently expressed in HeLa cells, showing significant colocalization with Rab11. While supportive of the hypothesis that the SH3TC genes encode a novel family of recycling endosome proteins, the targeting of SH3TC1 to this organelle does not appear to be Rab11-dependent (Supplementary Fig. 5A). Furthermore, the tissue expression pattern of SH3TC1, compared to the exclusive expression of SH3TC2 in Schwann cells, is yet to be determined and will require the development of specific antibodies and reagents.

### 3.5 SH3TC2 evolved in conjunction with the evolutionary appearance of Schwann cells and peripheral nerve myelination

**Fig. 6:**
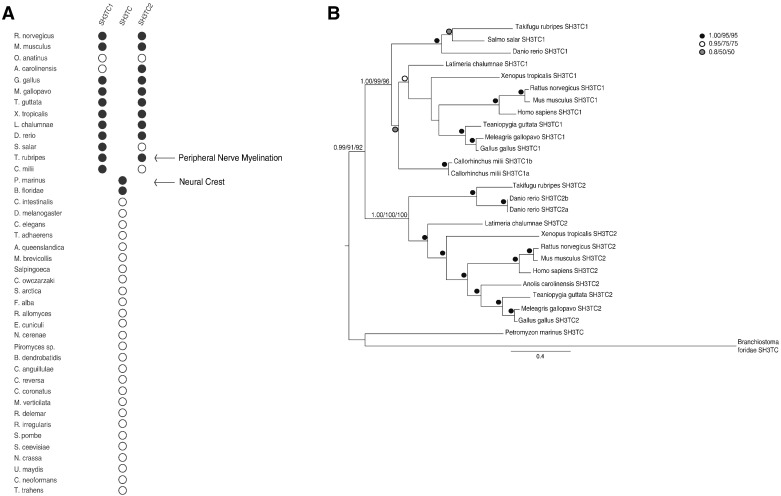
The evolution of the SH3TC homologues is concurrent with Schwann cell mediated myelination of peripheral nerve axons. A. Dot plot of comparative genomic analysis for SH3TC homologues in opisthokonts. Homologues of SH3TC2 and SH3TC1 were found in bony fish. Pre-duplicate SH3TC homologues (middle row) were also found in more basal chordates, but not in any taxa in the more basal groups of animals, holozoa or holomycetes. B. This phylogenetic analysis shows the duplication of the SH3TC gene prior to the jawed fish. The best Bayesian topology is shown, with the scale bar representing 0.4 changes per site. Node support values for key nodes are provided in the order of posterior probability, PhyML and RAxML bootstrap values. Nodes with support values greater than 0.8/50%/50% are symbolized as inset.

The exclusive expression of Sh3tc2 in myelinating Schwann cells points to a very specialized function and lends support to the hypothesis that SH3TC2 and Schwann cells might have co-evolved. Schwann cells are derived from the neural crest, a specialized population of multipotent migratory embryonic cells exclusive to vertebrates [35]. Furthermore, the myelination of axons by glia such as Schwann cells is also a relatively recent evolutionary development, appearing first in jawed vertebrates. The resulting energy-efficient and fast conduction of action potentials across significant distances associated with myelination is thought to have had obvious competitive advantages [36]. We therefore carried out a comprehensive evolutionary study of the *SH3TC2* gene, taking advantage of the extensive taxon sampling of genomic data now available for animals. Using this approach, we found that the *SH3TC2* gene is present in taxa from humans to bony fish (Actinopterygii), lending substantial support to our hypothesis that the *SH3TC2* gene emerged at the same time as Schwann cell-mediated peripheral nerve myelination (Fig. 6A and Supplementary Fig. 6).

Further analysis of available genomic data found that the *SH3TC1* gene is also present in metazoa, as basally as sharks. Additionally, single homologues of *SH3TC* genes that resolve as preduplicates to either *SH3TC1* and *SH3TC2* first appears in Branchiostoma (lancelet), thought to be representative of early chordates, and by the Hyperoatia (lampreys) (Fig. 6B). Interestingly, our data suggest that the two *SH3TC* genes 1 and 2 are derived from a gene duplication that occurred with the emergence of jawed fish, around the same time as when whole genome duplications are said to have played a pivotal role in the emergence of authentic neural crest cells and their specialized functions [37], [38], [39].

### 3.6 SH3TC2 associates with integrin-α_6_, highlighting a potential key role for Schwann cell Rab11-dependent maintenance of peripheral nerve myelination

All CMT4C-associated mutations in SH3TC2 disrupt the SH3TC2–Rab11 interaction [13]. Taken together with our expression and evolutionary data, we hypothesized that the maintenance of peripheral nerve myelination must be dependent on the SH3TC2–Rab11-mediated trafficking of specific plasma membrane receptors in Schwann cells. We therefore set out to identify potential SH3TC2 cargo proteins that might shed light on the pathogenesis of CMT4C.

To identify proteins that could associate with SH3TC2, we used the BioID technique [40], [41], [42]. This technique takes advantage of a promiscuous biotin ligase, BirA*, that labels proteins within an ~ 10 nm radius when fused to a protein of interest and expressed in cells [43]. Our objective was to identify cargo proteins expressed in the myelinated Schwann cell, where SH3TC2 is expressed in vivo. However, maintaining Schwann cells for prolonged periods of time in the myelinated state in culture was not technically possible. We therefore adopted a surrogate approach by stably expressed BirA*-SH3TC2 in a number of cell lines with the aim of identifying cargo proteins that might also be expressed in myelinated Schwann cells in vivo.

**Fig. 7:**
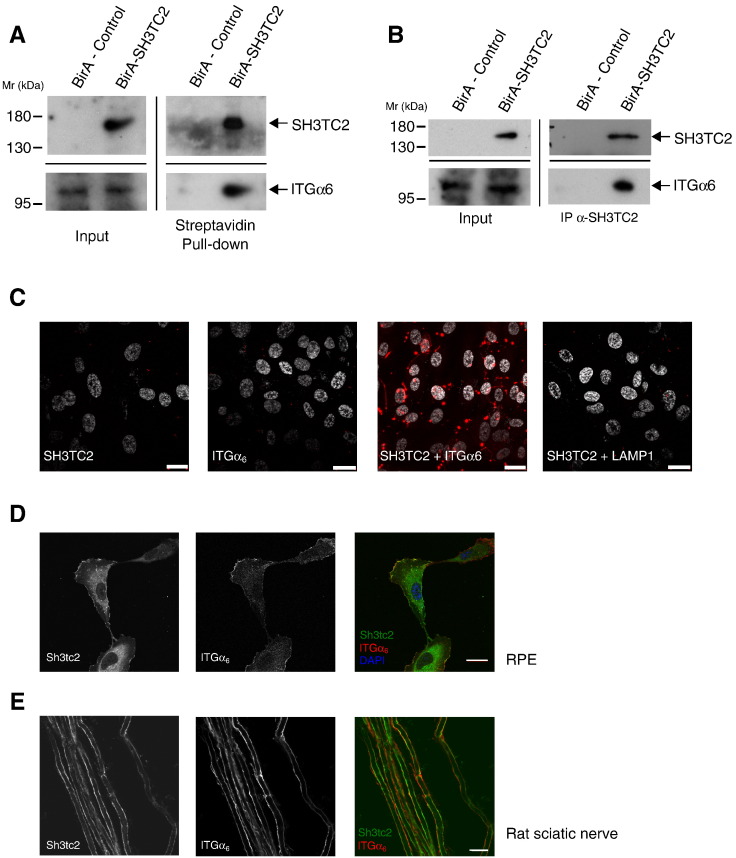
SH3TC2 associates with integrin-α_6_. A. Western blot of BioID experiment from RPE cells stably expressing either BirA alone or BirA-SH3TC2. Streptavidin was used to isolate biotinylated proteins before immunoblotting with antibodies towards SH3TC2 and integrin-α_6_ (ITGα6) (right panel). 1% of the cell lysate was used to verify expression of SH3TC2 and integrin-α_6_ in these cells (left panel). Note that a more sensitive chemiluminescent reagent (Supersignal West Femto) was required to detect integrin-α_6_ from RPE cell lysates and a number of additional bands were often seen using this method (see Supplementary Fig. 5B). In contrast, only a single band corresponding to integrin-α_6_ was seen in the BirA-SH3TC2 pull-down experiment, which could be visualized using the conventional chemiluminescence reagent (WesternBright). B. Immunoprecipitation of SH3TC2 from RPE cells stably-expressing either BirA alone or BirA-SH3TC2 using an anti-SH3TC2 antibody (2058). The right panel shows that integrin-α_6_ co-immunoprecipitates with SH3TC2. C. The proximity ligation assay was applied to RPE cells stably-expressing SH3TC2. Rolling circle amplification, detected by a red fluorescent probe, was only seen when cells were probed with both anti-SH3TC2 and anti-integrin-α_6_ (ITGα6) antibodies. No amplification was seen when either anti-SH3TC2 or anti-integrin-α_6_ antibodies were used alone, or in conjunction with antibodies towards the lysosomal marker protein, LAMP1, which acts as a negative control. Nuclei were stained with DAPI and are shown in white. Scale bar denotes 20 μm. D. Colocalization immunofluorescence study of RPE cells stably-expressing SH3TC2 by confocal microscopy. SH3TC2 and integrin-α_6_ were visualized using antibodies towards SH3TC2 (2058) and integrin-α_6_ (ITGα6), respectively. Scale bar denotes 10 μm. E. Colocalization immunofluorescence study of rat sciatic nerve teased fibers by confocal microscopy. SH3TC2 and integrin-α_6_ were visualized using antibodies towards SH3TC2 (2058) and integrin-α_6_ (ITGα6), respectively. Scale bar denotes 10 μm.

Using this approach, we identified integrin-α_6_ as an SH3TC2-associated protein in Retinal Pigment Epithelial cells (RPE) (Fig. 7A). Significantly, integrin-α_6_, along with other integrins expressed in Schwann cells, has previously been shown to play an important role in peripheral nerve myelination [5], [6], [44], [45]. Of note, integrin-α_6_ was not identified from HeLa cells and the immortalized Schwann cell line, IFRS-1, which can be explained by the undetectable levels of integrin-α_6_ expression in these cells (Supplementary Fig. 5B). The association between SH3TC2 and integrin-α_6_ was verified using two complementary techniques: co-immunoprecipitation of SH3TC2 and integrin-α_6_ from RPE cells stably-expressing SH3TC2 (Fig. 7B), and the Duolink proximity ligation assay [46] (Fig. 7C). Consistent with these data, immunofluorescence microscopy revealed a high degree of colocalization between stably-expressed SH3TC2 and endogenous integrin-α_6_ in RPE cells, being particularly concentrated at the plasma membrane (Fig. 7D). Furthermore, endogenously expressed SH3TC2 and integrin-α_6_ were also found to colocalize in teased sciatic-nerve fibers (Fig. 7E).

## 4 Discussion

The exclusive expression of the Sh3tc2 protein in myelinating Schwann cells explains the demyelinating peripheral neuropathy that characterizes CMT4C. Moreover, having shown that Sh3tc2 expression increases as myelination proceeds and, conversely, that Sh3tc2 is downregulated following nerve injury and demyelination, our data point strongly to a role for Sh3tc2 in the maintenance of the peripheral nerve myelin sheath. While previous studies have also proposed that Sh3tc2 is expressed in peripheral nerves and Schwann cells [11], [29], [47], the supporting evidence has been based on RNA expression studies and the expression of GFP protein driven by the Sh3tc2 promoter in the Sh3tc2 knock-out mouse. Our study is the first to characterize the spatial along with the temporal expression pattern of the endogenous Sh3tc2 protein in vivo. It is worth noting that the recently published human protein atlas (www.proteinatlas.org) [48] states that the SH3TC2 protein is expressed in a wide variety of tissues, contrary to our data. However, the antibody used by Uhlen et al. detects a protein band closer to 70 kDa by western blotting, rather than the predicted 144 kDa band that we see in our study, and does not detect exogenously-expressed SH3TC2 constructs (either by western blotting or immunofluorescence microscopy) in our hands (data not shown), which suggests that non-specificity of the antibody is the most likely explanation for this discrepancy in this case. Furthermore, the available transcriptomic and RNA sequencing tissue expression databases show inconsistent results with respect to SH3TC2 expression across 8 human and 24 mouse experiments. Nevertheless, and consistent with our findings, SH3TC2 RNA expression was not detected in most tissues analyzed (https://www.ebi.ac.uk/gxa/home). Additionally, Senderek et al. have previously reported the detection of SH3TC2 RNA transcripts in brain, spinal cord, skeletal muscle, in addition to sciatic nerve, by northern blotting and RT-PCR [11]. However, multiple alternatively-spliced transcripts were found to predominate in brain and spinal cord, predicted to cause extensive truncations and resulting protein degradation if translated, which would also be entirely consistent with our failure to detect SH3TC2 at the protein level in these tissues.

Sh3tc2 expression is upregulated late during myelination, becoming prominent at 32 days after birth, and appears to follow the upregulation of its binding partner, Rab11. These data also complement the findings of Arnaud et al. who previously developed and characterized an Sh3tc2 knock-out mouse as a disease model for CMT4C [29], [49]. Significantly, these mice developed normally, and neurophysiological, ultrastructural and phenotypic abnormalities were reported only after 1 month, 2 months and 6 months, respectively. Furthermore, a recent report revealed two cis-acting regulatory elements upstream from the *SH3TC2* gene that are responsive to the transcription factor SOX10, which is known to play a crucial role in peripheral nerve myelination [47]. Taken together with our data, we conclude that SH3TC2 is not required for myelination per se but is required to maintain the structure and integrity of the myelin sheath once it is formed. The absence of Sh3tc2 in these mice (and in CMT4C patients) eventually leads to abnormalities such as widening of the node of Ranvier, a critical structure containing a high concentration of voltage-gated sodium channels necessary for the efficient propagation of action potentials [50].

Our evolutionary data also support a specific role for SH3TC2 in peripheral nerve myelination. Schwann cells are derived from neural crest cells, a group of multi-potent cells closely associated with vertebrate evolution. Furthermore, although many diverse organisms contain nervous systems that include supporting cells analogous to glia, the existence of Schwann-cell mediated myelinated axons occurs only with the emergence of the gnathostomata [37]. By searching the current diversity of animal genome DNA databases, we found clear homologues to SH3TC2 only in bony vertebrates, consistent with the findings of Senderek et al. (who had access to only a limited number of completed genomes at that time) [11]. Interestingly, and despite possessing fully formed peripheral nerve myelin, we failed to find a clear orthologue of the *SH3TC2* gene in cartilaginous vertebrates such as sharks. This could be explained by incomplete coverage in the currently available shark genome or, as a highly speculative but intriguing alternative, may suggest that these species might have evolved an alternative strategy that could include the use of one of the three *SH3TC1* genes that we identified (Fig. 6B and Supplementary Fig. 6), to maintain the integrity of their peripheral nerve myelin sheaths.

In contrast to SH3TC2, many demyelinating CMT-associated proteins that are known or predicted to play a role in intracellular membrane trafficking, such as LITAF, MTMR2/MTMR13, NDRG1, FIG4, and DNM2, are widely expressed in different tissues [51], [52], [53], [54], [55] and are also evolutionarily conserved in species that predate the emergence of chordates [56], [57], [58], [59], [60]. These proteins therefore possess diverse general membrane trafficking functions applicable across different tissues but must also have critical non-redundant roles in maintaining peripheral nerve function.

Our data, taken together with information from genome databases, strongly suggest that SH3TC2 evolved specifically to play a unique, Rab11-dependent role that is exclusive to myelinated Schwann cells. Moreover, we can also conclude that the structural integrity of the myelin sheath is dependent on the expression of SH3TC2 in Schwann cells along with its ability to associate with Rab11. This leads to the hypothesis that the maintenance of peripheral nerve myelination must be reliant on the SH3TC2/Rab11-mediated intracellular trafficking of a specific Schwann cell cargo. Here, we show that SH3TC2 associates with integrin-α_6_, a crucial laminin receptor expressed on the basolateral surface of Schwann cells and known to play an important role in peripheral nerve myelination [7]. Indeed, integrin-α_6_ in conjunction with integrin-β_4_ is expressed later in myelination, similarly to SH3TC2, and loss of this heterodimeric laminin receptor is associated with progressive myelin structural instability [6]. Integrins, including the integrin-α_6_/β_4_ heterodimer, are known to undergo Rab11-dependent endocytic recycling, which leads to the intriguing possibility that SH3TC2 mediates the Rab11-dependent trafficking of integrin-α_6_/β_4_ in myelinating Schwann cells in vivo. These data lead us therefore to speculate that loss of the SH3TC2–Rab11 interaction, as a consequence of CMT4C-associated pathogenic mutations, could cause aberrant endocytic recycling of integrin-α_6_/β_4_, leading to the loss of attachment between the Schwann cell abaxonal membrane and the extracellular matrix, resulting in the destabilization of the myelin sheath and, eventually, the demyelinating peripheral neuropathy that characterizes CMT4C. Nevertheless, how SH3TC2 and integrin-α_6_ interact precisely at the molecular level, and whether SH3TC2 also regulates the trafficking of other Schwann cell cargo proteins, has yet to be determined. Furthermore, a key unanswered question is whether the pathological mechanisms underlying more than one subtype of demyelinating CMT can be linked to the endocytic trafficking of a common Schwann cell cargo, such as integrin-α_6_/β_4_, which would have profound implications for the developments of future treatments.

## 5 Conclusions

We conclude that SH3TC2 is an exclusive and specific Schwann cell protein that is expressed late in peripheral nerve myelination, pointing to a key role in maintaining the structural integrity of peripheral nerve myelin sheaths. We speculate that this function is mediated through an interaction between SH3TC2 and integrin-α_6_ which regulates the Rab11-dependent endocytic recycling of this key Schwann cell surface receptor. Our expression data help explain why mutations in SH3TC2, previously shown to inhibit Rab11 binding, lead specifically to the demyelinating peripheral neuropathy that characterizes CMT4C. In the absence of any current treatments and the relatively late expression of SH3TC2 in myelination, our findings therefore highlight a potential temporal therapeutic window where future treatments might be targeted.

The following are the supplementary data related to this article.

Supplementary figures Image 1

Table S1 Table S1

## Transparency Document

Transparency document. Image 2

## References

[1] Reilly M.M., Murphy S.M., Laura M. (2011). Charcot-Marie-Tooth disease. J. Peripher. Nerv. Syst..

[2] Rossor A.M., Polke J.M., Houlden H., Reilly M.M. (2013). Clinical implications of genetic advances in Charcot-Marie-Tooth disease. Nat. Rev. Neurol..

[3] Harding A.E., Thomas P.K. (1980). The clinical features of hereditary motor and sensory neuropathy types I and II. Brain.

[4] Buttermore E.D., Thaxton C.L., Bhat M. a (2013). Organization and maintenance of molecular domains in myelinated axons. J. Neurosci. Res..

[5] Berti C., Nodari A., Wrabetz L., Feltri M.L. (2006). Role of integrins in peripheral nerves and hereditary neuropathies. NeuroMolecular Med..

[6] Nodari A., Previtali S.C., Dati G., Occhi S., Court F. a, Colombelli C. (2008). Alpha6beta4 integrin and dystroglycan cooperate to stabilize the myelin sheath. J. Neurosci..

[7] Previtali S.C., Nodari A., Taveggia C., Pardini C., Dina G., Villa A. (2003). Expression of laminin receptors in schwann cell differentiation: evidence for distinct roles. J. Neurosci..

[8] De Franceschi N., Hamidi H., Alanko J., Sahgal P., Ivaska J. (2015). Integrin traffic - the update. J. Cell Sci..

[9] Bretscher M.S. (1992). Circulating integrins: alpha 5 beta 1, alpha 6 beta 4 and Mac-1, but not alpha 3 beta 1, alpha 4 beta 1 or LFA-1. EMBO J..

[10] Yoon S.O., Shin S., Mercurio A.M. (2005). Hypoxia stimulates carcinoma invasion by stabilizing microtubules and promoting the Rab11 trafficking of the 6 4 integrin. Cancer Res..

[11] Senderek J., Bergmann C., Stendel C., Kirfel J., Verpoorten N., De Jonghe P. (2003). Mutations in a gene encoding a novel SH3/TPR domain protein cause autosomal recessive Charcot-Marie-Tooth type 4C neuropathy. Am. J. Hum. Genet..

[12] Lupski J.R., Reid J.G., Gonzaga-Jauregui C., Rio Deiros D., Chen D.C., Nazareth L. (2010). Whole-genome sequencing in a patient with Charcot-Marie-Tooth neuropathy. N. Engl. J. Med..

[13] Roberts R.C., Peden A.A., Buss F., Bright N.A., Latouche M., Reilly M.M. (2010). Mistargeting of SH3TC2 away from the recycling endosome causes Charcot-Marie-Tooth disease type 4C. Hum. Mol. Genet..

[14] Stendel C., Roos A., Kleine H., Arnaud E., Ozcelik M., Sidiropoulos P.N. (2010). SH3TC2, a protein mutant in Charcot-Marie-Tooth neuropathy, links peripheral nerve myelination to endosomal recycling. Brain.

[15] Lupo V., Galindo M.I., Martínez-Rubio D., Sevilla T., Vílchez J.J., Palau F. (2009). Missense mutations in the SH3TC2 protein causing Charcot-Marie-Tooth disease type 4C affect its localization in the plasma membrane and endocytic pathway. Hum. Mol. Genet..

[16] Shea D.K., Walsh C.J. (1987). mRNAs for alpha- and beta-tubulin and flagellar calmodulin are among those coordinately regulated when *Naegleria gruberi* amebae differentiate into flagellates. J. Cell Biol..

[17] van den Ent F., Löwe J. (2006). RF cloning: a restriction-free method for inserting target genes into plasmids. J. Biochem. Biophys. Methods.

[18] Bond S.R., Naus C.C. (2012). RF-cloning.org: an online tool for the design of restriction-free cloning projects.. Nucleic Acids Res..

[19] Kaewkhaw R., Scutt A.M., Haycock J.W. (2012). Integrated culture and purification of rat Schwann cells from freshly isolated adult tissue. Nat. Protoc..

[20] Eaton S.L., Roche S.L., Llavero Hurtado M., Oldknow K.J., Farquharson C., Gillingwater T.H. (2013). Total protein analysis as a reliable loading control for quantitative fluorescent western blotting. PLoS ONE.

[21] Edgar R.C. (2004). MUSCLE: multiple sequence alignment with high accuracy and high throughput. Nucleic Acids Res..

[22] Darriba D., Taboada G.L., Doallo R., Posada D. (2011). ProtTest 3: fast selection of best-fit models of protein evolution. Bioinformatics.

[23] Guindon S., Gascuel O. (2003). A simple, fast, and accurate algorithm to estimate large phylogenies by maximum likelihood. Syst. Biol..

[24] Lartillot N., Lepage T., Blanquart S. (2009). PhyloBayes 3: a Bayesian software package for phylogenetic reconstruction and molecular dating. Bioinformatics.

[25] Stamatakis A. (2006). RAxML-VI-HPC: maximum likelihood-based phylogenetic analyses with thousands of taxa and mixed models. Bioinformatics.

[26] Felsenstein J. (2005). PHYLIP (Phylogeny Inference Package) version 3.6. Distributed by the Author. Department of Genome Sc.

[27] Gordon D.E., Mirza M., Sahlender D. a, Jakovleska J., Peden A. a (2009). Coiled-coil interactions are required for post-Golgi R-SNARE trafficking. EMBO Rep..

[28] Sherman D.L., Wu L.M.N., Grove M., Gillespie C.S., Brophy P.J. (2012). Drp2 and periaxin form Cajal bands with dystroglycan but have distinct roles in Schwann cell growth. J. Neurosci..

[29] Arnaud E., Zenker J., de Preux Charles A.-S.S., Stendel C., Roos A., Medard J.J. (2009). SH3TC2/KIAA1985 protein is required for proper myelination and the integrity of the node of Ranvier in the peripheral nervous system. Proc. Natl. Acad. Sci. U. S. A..

[30] L. Morgan, K. Jessen, R. Mirsky, The effects of cAMP on differentiation of cultured Schwann cells: progression from an early phenotype (04 +) to a myelin phenotype (P0 +, GFAP −, N-CAM −, NGF −, J. Cell Biol. 112 (1991) 457–467. (http://jcb.rupress.org/content/112/3/457.abstract (accessed November 24, 2014)). 10.1083/jcb.112.3.457 PMC2288828 1704008

[31] Chen Z.-L., Yu W.-M., Strickland S. (2007). Peripheral regeneration. Annu. Rev. Neurosci..

[32] Arthur-Farraj P.J., Latouche M., Wilton D.K., Quintes S., Chabrol E., Banerjee A. (2012). c-Jun reprograms Schwann cells of injured nerves to generate a repair cell essential for regeneration. Neuron.

[33] Thomson C.E., Griffiths I.R., McCulloch M.C., Kyriakides E., Barrie J. a, Montague P. (1993). In vitro studies of axonally-regulated Schwann cell genes during Wallerian degeneration. J. Neurocytol..

[34] Jung J., Cai W., Lee H.K., Pellegatta M., Shin Y.K., Jang S.Y. (2011). Actin polymerization is essential for myelin sheath fragmentation during Wallerian degeneration. J. Neurosci..

[35] Jessen K.R., Mirsky R. (2005). The origin and development of glial cells in peripheral nerves. Nat. Rev. Neurosci..

[36] Hartline D.K., Colman D.R. (2007). Rapid conduction and the evolution of giant axons and myelinated fibers. Curr. Biol..

[37] Zalc B., Colman D.R. (2000). Origins of vertebrate success. Science.

[38] Dehal P., Boore J.L. (2005). Two rounds of whole genome duplication in the ancestral vertebrate. PLoS Biol..

[39] Green S. a, Bronner M.E. (2013). Gene duplications and the early evolution of neural crest development. Semin. Cell Dev. Biol..

[40] Roux K.J., Kim D.I., Raida M., Burke B. (2012). A promiscuous biotin ligase fusion protein identifies proximal and interacting proteins in mammalian cells. J. Cell Biol..

[41] Roux K.J., Kim D.I., Burke B. (2013). BioID: A Screen for Protein–Protein Interactions.

[42] Morriswood B., Havlicek K., Demmel L., Yavuz S., Sealey-Cardona M., Vidilaseris K. (2013). Novel bilobe components in *Trypanosoma brucei* identified using proximity-dependent biotinylation. Eukaryot. Cell..

[43] Kim D.I., Kc B., Zhu W., Motamedchaboki K., Doye V., Roux K.J. (2014). Probing nuclear pore complex architecture with proximity-dependent biotinylation. Proc. Natl. Acad. Sci. U. S. A..

[44] Pellegatta M., De Arcangelis a., D'Urso a., Nodari a., Zambroni D., Ghidinelli M. (2013). α6β1 and 7 1 integrins are required in Schwann cells to sort axons. J. Neurosci..

[45] Niessen C.M., Cremona O., Daams H., Ferraresi S., Sonnenberg A., Marchisio P.C. (1994). Expression of the integrin alpha 6 beta 4 in peripheral nerves: localization in Schwann and perineural cells and different variants of the beta 4 subunit. J. Cell Sci..

[46] Söderberg O., Gullberg M., Jarvius M., Ridderstråle K., Leuchowius K.-J., Jarvius J. (2006). Direct observation of individual endogenous protein complexes in situ by proximity ligation. Nat. Methods.

[47] Brewer M.H., Ma K.H., Beecham G.W., Gopinath C., Baas F., Choi B.-O. (2014). Haplotype-specific modulation of a SOX10/CREB response element at the Charcot-Marie-Tooth disease type 4C locus SH3TC2. Hum. Mol. Genet..

[48] Uhlén M., Fagerberg L., Hallström B.M., Lindskog C., Oksvold P., Mardinoglu A. (2015). Tissue-based Map of the Human Proteome.

[49] Gouttenoire E.A., Lupo V., Calpena E., Bartesaghi L., Schüpfer F., Médard J.-J. (2013). Sh3tc2 Deficiency Affects Neuregulin-1/ErbB Signaling, Glia.

[50] Poliak S., Peles E. (2003). The local differentiation of myelinated axons at nodes of Ranvier. Nat. Rev. Neurosci..

[51] Moriwaki Y., Begum N.A., Kobayashi M., Matsumoto M., Toyoshima K., Seya T. (2001). *Mycobacterium bovis* Bacillus Calmette-Guerin and its cell wall complex induce a novel lysosomal membrane protein, SIMPLE, that bridges the missing link between lipopolysaccharide and p53-inducible gene, LITAF(PIG7), and estrogen-inducible gene, EET-1. J. Biol. Chem..

[52] Bolino A., Marigo V., Ferrera F., Loader J., Romio L., Leoni A. (2002). Molecular characterization and expression analysis of Mtmr2, mouse homologue of MTMR2, the myotubularin-related 2 gene, mutated in CMT4B. Gene.

[53] Lachat P., Shaw P., Gebhard S., Van Belzen N., Chaubert P., Bosman F.T. (2002). Expression of NDRG1, a differentiation-related gene, in human tissues. Histochem. Cell Biol..

[54] Vaccari I., Carbone a., Previtali S.C., Mironova Y.a., Alberizzi V., Noseda R. (2014). Loss of Fig4 in both Schwann cells and motor neurons contributes to CMT4J neuropathy. Hum. Mol. Genet..

[55] Cook T. a, Urrutia R., McNiven M. a (1994). Identification of dynamin 2, an isoform ubiquitously expressed in rat tissues. Proc. Natl. Acad. Sci. U. S. A..

[56] Wang P.H., Wan D.H., Pang L.R., Gu Z.H., Qiu W., Weng S.P. (2012). Molecular cloning, characterization and expression analysis of the tumor necrosis factor (TNF) superfamily gene, TNF receptor superfamily gene and lipopolysaccharide-induced TNF-alpha factor (LITAF) gene from Litopenaeus vannamei. Dev. Comp. Immunol..

[57] Laporte J., Blondeau F., Buj-Bello A., Tentler D., Kretz C., Dahl N. (1998). Characterization of the myotubularin dual specificity phosphatase gene family from yeast to human. Hum. Mol. Genet..

[58] Melotte V., Qu X., Ongenaert M., van Criekinge W., de Bruïne A.P., Baldwin H.S. (2010). The N-myc downstream regulated gene (NDRG) family: diverse functions, multiple applications. FASEB J..

[59] Gary J.D., Sato T.K., Stefan C.J., Bonangelino C.J., Weisman L.S., Emr S.D. (2002). Regulation of Fab1 phosphatidylinositol 3-phosphate 5-kinase pathway by Vac7 protein and Fig4, a polyphosphoinositide phosphatase family member. Mol. Biol. Cell.

[60] Smaczynska-de Rooij I.I., Allwood E.G., Aghamohammadzadeh S., Hettema E.H., Goldberg M.W., Ayscough K.R. (2010). A role for the dynamin-like protein Vps1 during endocytosis in yeast. J. Cell Sci..

