# Supplementary material for: Exclusive expression of the Rab11 effector SH3TC2 in Schwann cells links integrin-α6 and myelin maintenance to Charcot-Marie-Tooth disease type 4C
Source: Biochim Biophys Acta. 2016 Jul;1862(7):1279–90. doi: 10.1016/j.bbadis.2016.04.003 (PMC4879868; doi:10.1016/j.bbadis.2016.04.003)
Supplement: Supplementary file 1 — Supplementary figures [file mmc1.pdf]

Supplementary figure 1

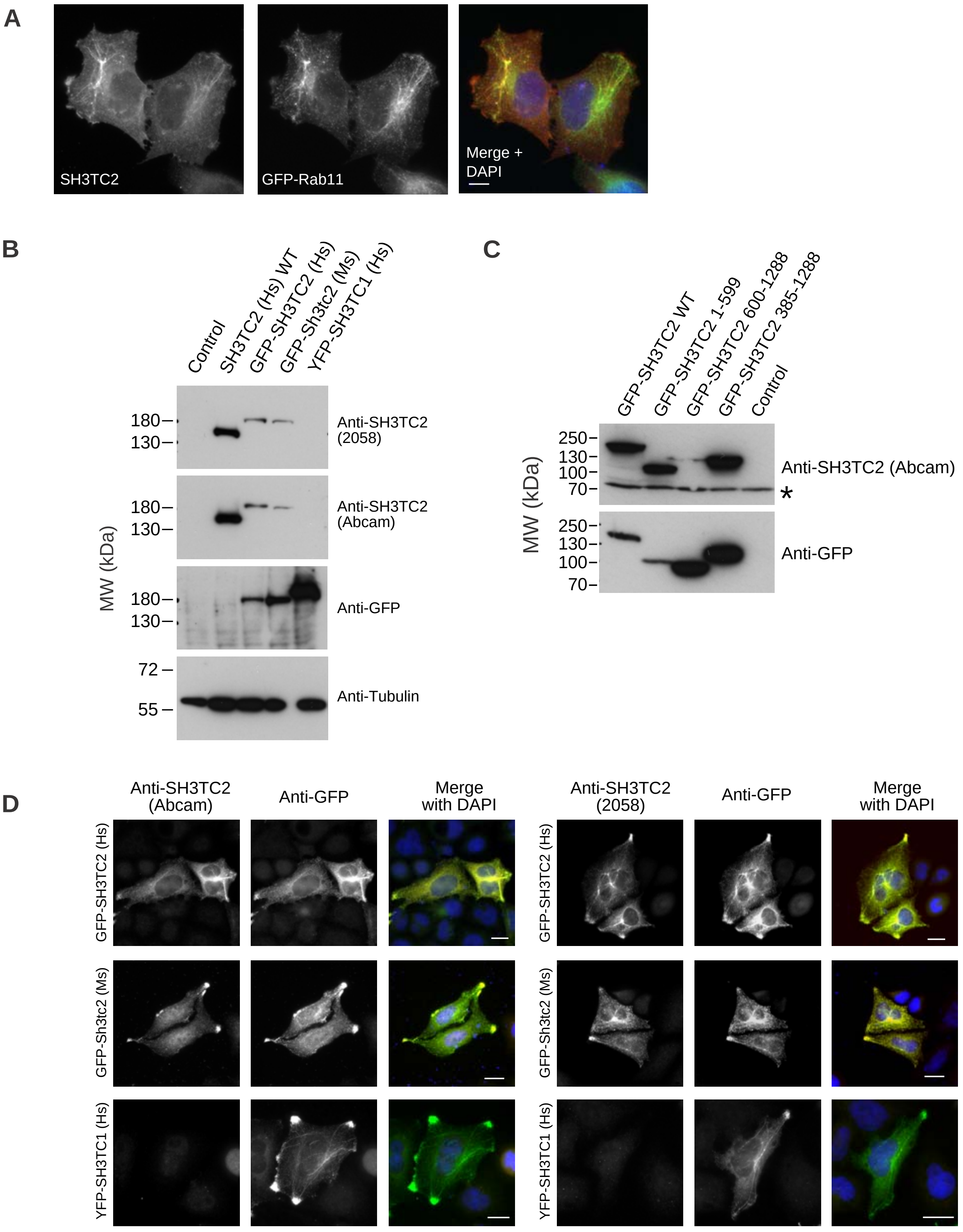

Supplementary figure 1

(A) Immunofluorescence microscopy showing that the polyclonal anti-SH3TC2 antibody (Abcam) recognises transiently-expressed human SH3TC2 in HeLa cells, colocalising with stably-expressed GFP-Rab11, which targets to the endocytic recycling compartment. Scale bar denotes 10 µm. (B) Western blot showing that the polyclonal anti-SH3TC2 antibodies (Abcam + 2058) recognise both human SH3TC2 and mouse Sh3tc2 proteins transiently expressed in HeLa cells. Note that the un-tagged SH3TC2 protein runs at the predicted molecular weight of 144 kDa. In contrast, endogenous SH3TC2 is not expressed in control HeLa cells. Furthermore, the SH3TC2 antibodies do not cross-react with SH3TC1. Note that the GFP antibody cross-reacts with YFP. (C) Western blot showing that the anti-SH3TC2 antibody (Abcam) recognises an epitope on SH3TC2 between residues 385 and 599. GFP-tagged constructs of SH3TC2 were transiently expressed in HeLa cells and the resulting lysates probed with the anti-SH3TC2 antibody (top panel) and an anti-GFP antibody to confirm expression (lower panel). '*' denotes a non-specific band that it occasionally seen in HeLa cells with the Abcam antibody. (D) Immunofluorescence microscopy confirming that both antibodies towards SH3TC2 (Abcam and 2058) recognise human SH3TC2 and mouse Sh3tc2 transiently expressed as GFP-tagged proteins in HeLa cells. In contrast, neither antibody cross reacts with YFP-SH3TC1 when transiently expresed in HeLa cells. Scale bars denote 20 µm.

Supplementary figure 2

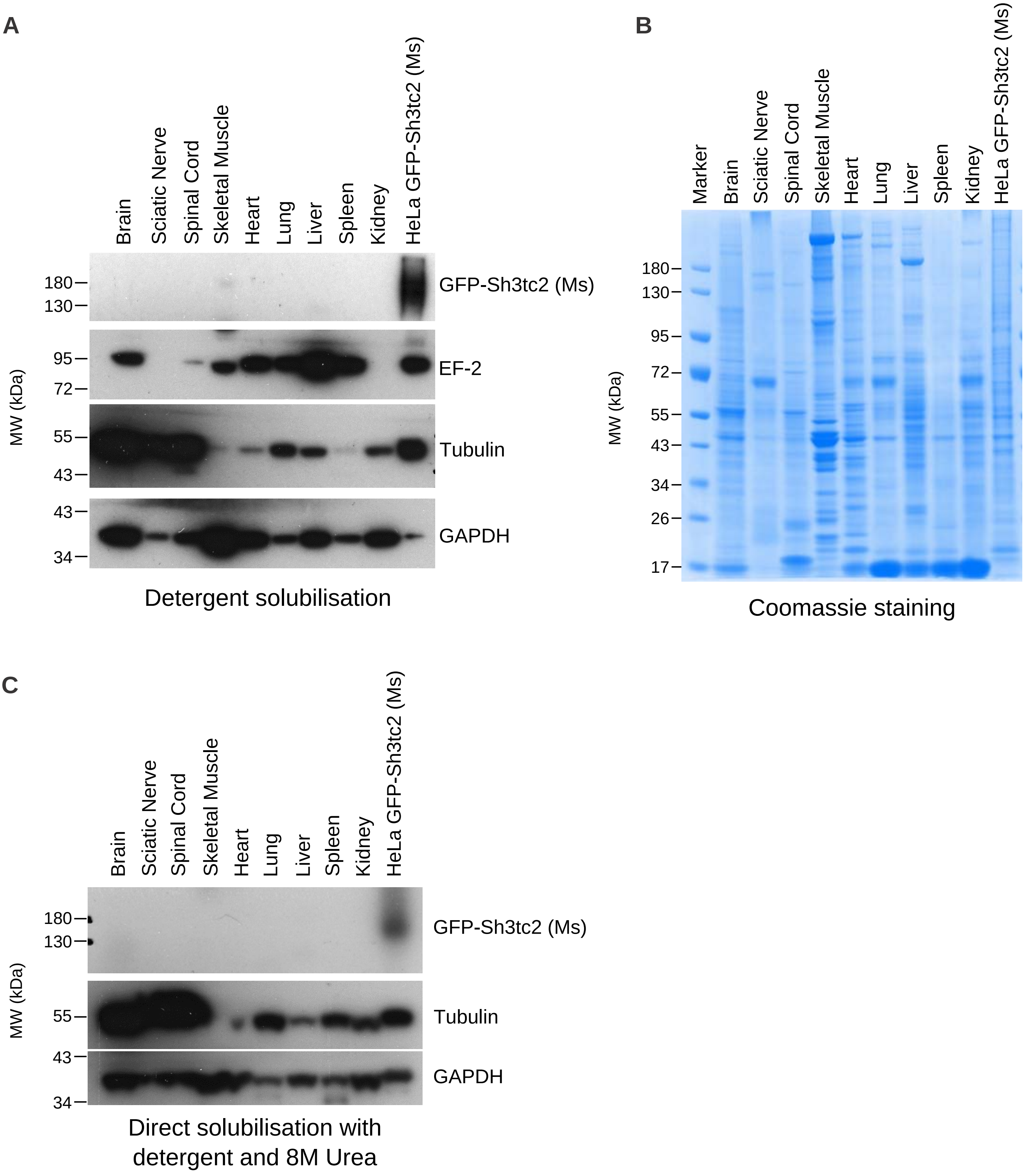

Supplementary figure 2

(A) Western blot of rat tissue lysates directly solubilised with RIPA buffer (50mM Tris pH 7.4, 150mM NaCl, 1mM EDTA, 1% Ipegal, 0.5% Sodium deoxycholate, 0.1% SDS and Complete protease inhibitor (Roche)). The protein concentration of each tissue lysate was quantified using the Precision Red Advanced Protein Assay (Cytoskeleton, Inc), mixed with LDS sample buffer and 30 µg loaded into each well of a 4-12% NuPAGE Bis-Tris gel before separation by electrophoresis in MOPS buffer (Thermo Fisher Scientific) and transfer to PVDF membranes. The membranes were probed for Sh3tc2 and the marker proteins, EF-2, α-tubulin and GAPDH. A cell lysate prepared from HeLa cells expressing GFP-Sh3tc2 (Ms) was used as a positive control. Using this approach, no expression of endogenous Sh3tc2 was detected from the rat tissues. A very faint band was seen in the skeletal muscle lane, which we consider to be non-specific due to its high molecular weight (>180 kDa) and inconsistent presence. (B) 30 µg of protein from each tissue lysate was loaded into wells of a 4-12% Bis-Tris gel before separation by electrophoresis and stained with InstantBlue protein stain (Sigma) to illustrate the relative protein loading used in (A). (C) Western blot of rat tissues solubilised directly with RIPA buffer also containing 8M Urea. 30 µg of protein was loaded into each well of a 4-12% NuPAGE Bis-Tris gel before separation by electrophoresis in MOPS buffer (Thermo Fisher Scientific) and transfer to PVDF membranes. The membranes were probed for Sh3tc2 and the marker proteins α-tubulin and GAPDH. A cell lysate prepared from HeLa cells expressing GFP-Sh3tc2 (Ms) was used as a positive control. Despite the fact that Sh3tc2 was detected following resuspension of the isolated detergent-insoluble pellet from sciatic nerves with 8M Urea (Fig. 1B), Sh3tc2 could not be detected following direct solubilisation of rat tissues in RIPA buffer containing 8M Urea.

Supplementary figure 3

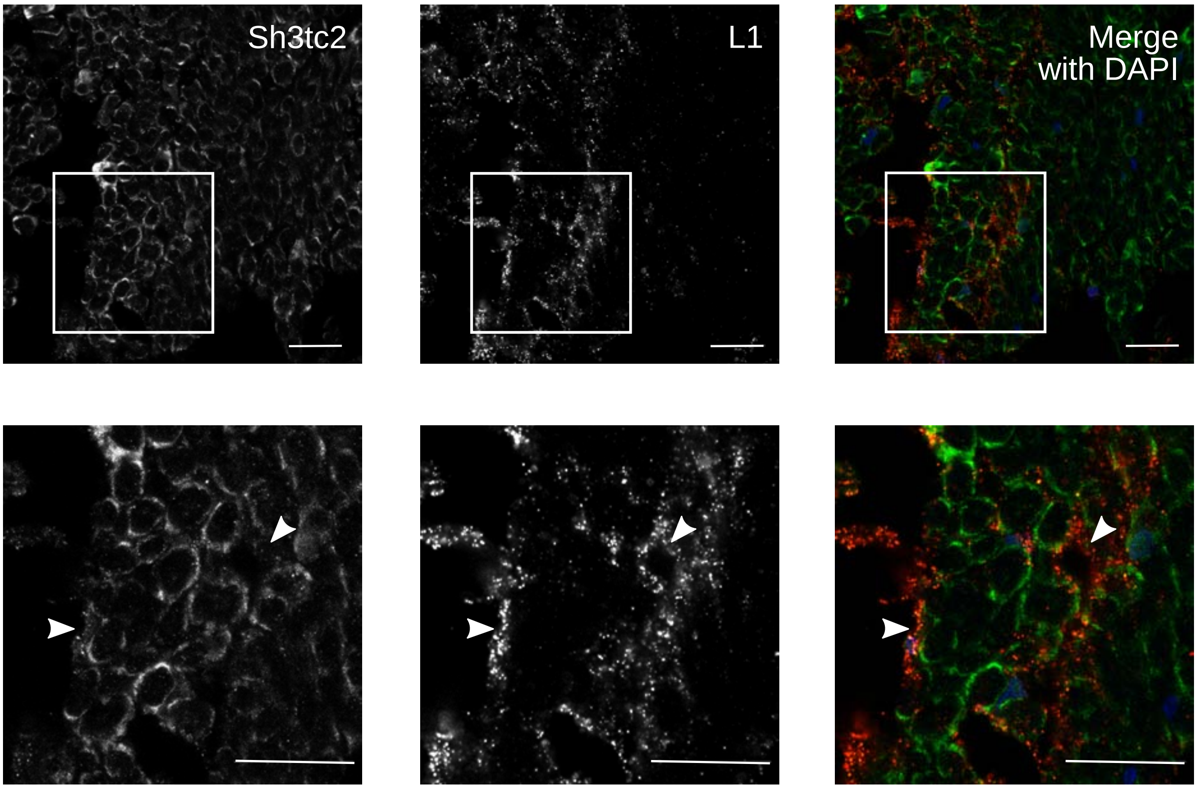

Supplementary figure 3
A transverse section of rat sciatic nerve showing that Sh3tc2 (Green) is not found in Remak cells labelled with L1 (Red). Magnified images of the areas denoted by the white squares are shown in the lower panels. Arrowheads denote areas of L1 staining with minimal staining for Sh3tc2. Scale bars denote 20 µm.

Supplementary figure 4

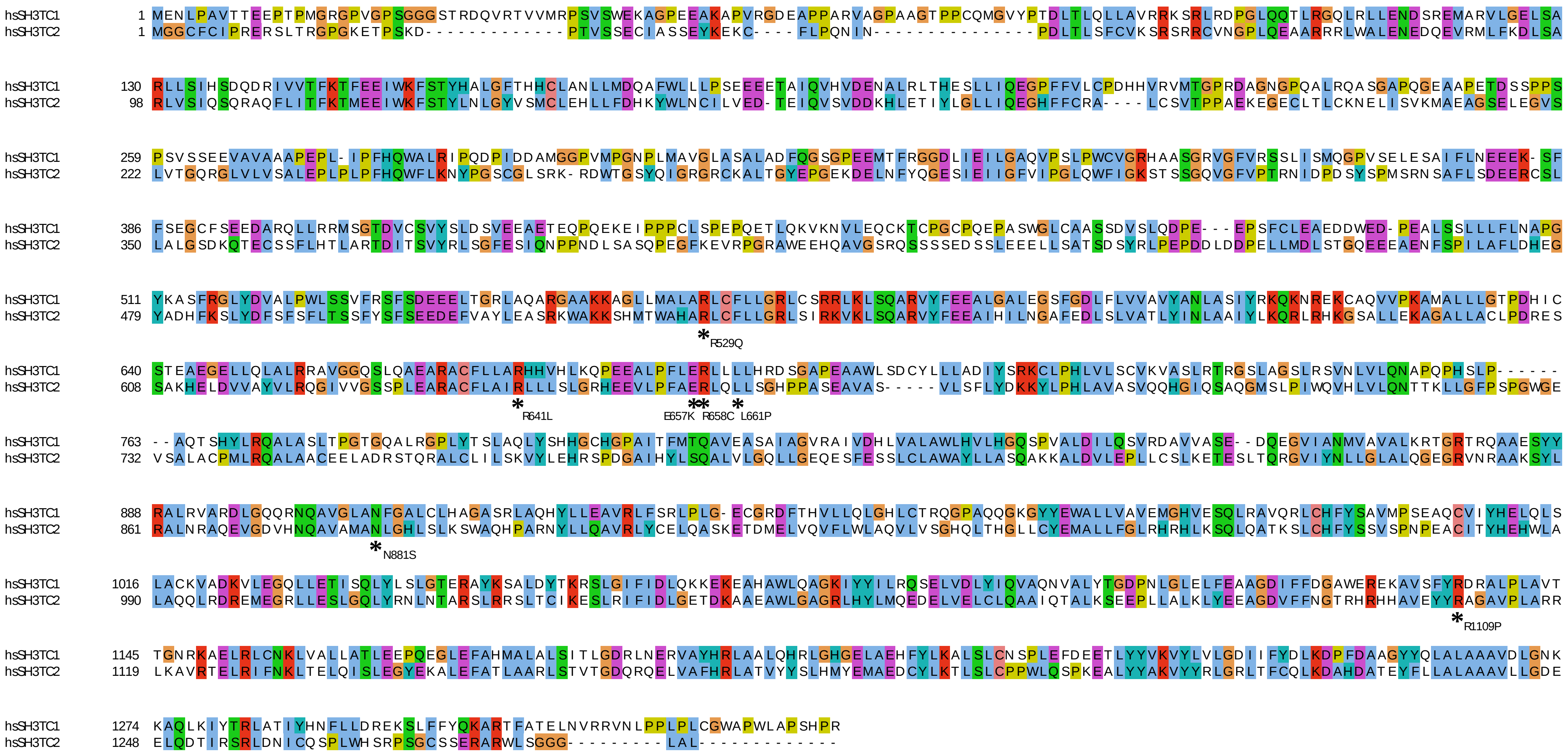

Supplementary figure 4

Alignment of amino acid sequences of human SH3TC1 and human SH3TC2 with the ClustalX default colourscheme applied. CMT4C-associated residues are indicated by ‘*’.

Supplementary figure 5

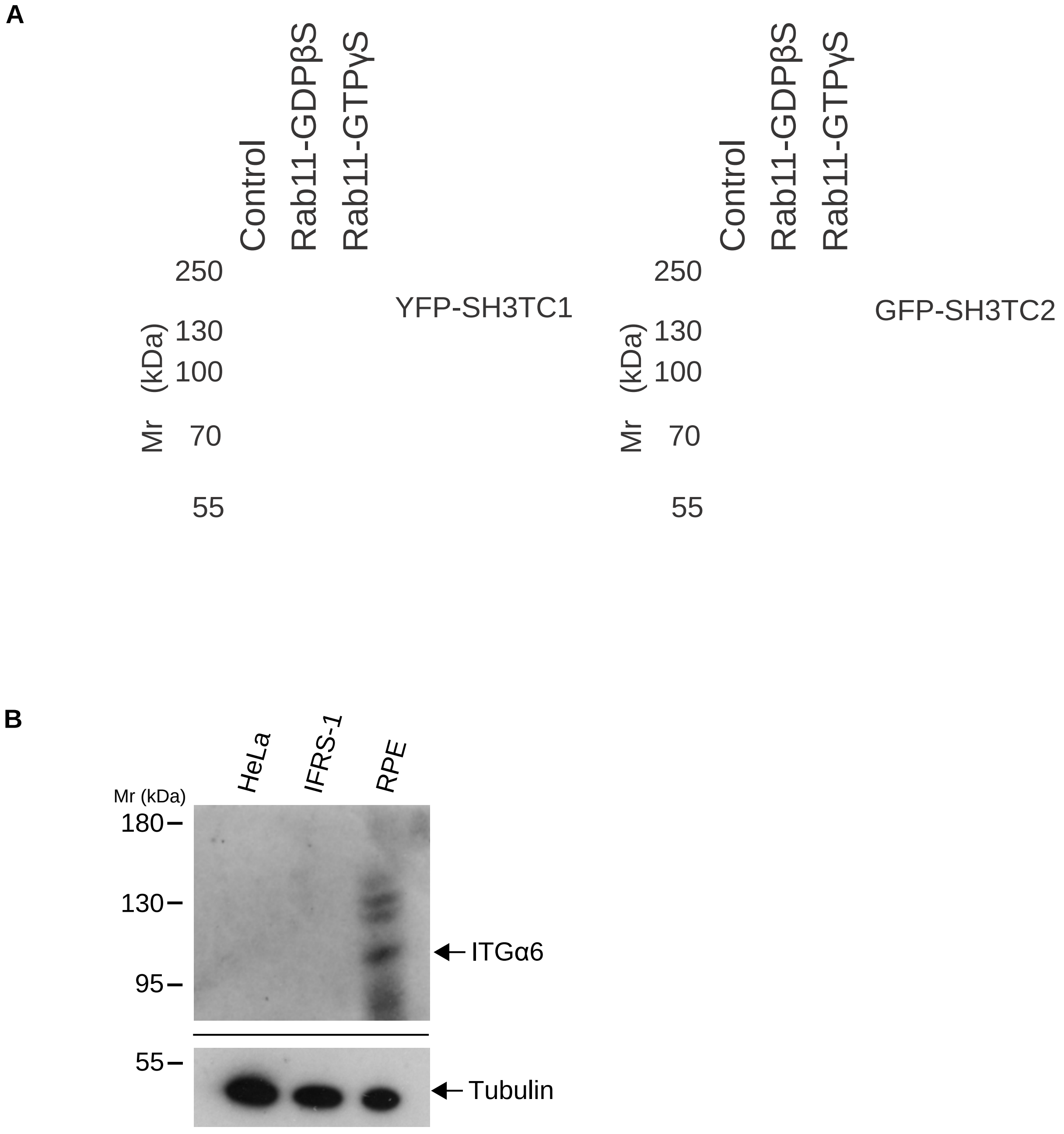

Supplementary figure 5
(A) Western blots of GST-Rab11 pull down experiments using lysates from HeLa cells transiently expressing YFP-SH3TC1 or GFP-SH3TC2. Glutathione sepharose beads without protein were used as controls. YFP-SH3TC1 or GFP-SH3TC2 were transiently expressed in HeLa cells for 48 hours following transfection of plasmid DNA. Cell lysates were then prepared followed by GST-Rab11 pull-down assays as previously described (Roberts et al. (2010), including pre-incubation with GDPβS or GTPγS (Sigma-Aldrich). Isolated proteins were separated by 7.5% SDS-PAGE and transferred to nitrocellulose membrane for western blotting and detection by chemiluminescence. (B) Intergrin-α6 is expressed in RPE cells but not in HeLa or IFRS-1 cells as shown by western blotting. Note that a more sensitive chemiluminescence reagent is required to detect expression of integrin-α6 (Supersignal West Femto, Thermo Fisher) and that multiple bands, in addition to ITGα6 at MW 119 kDa, were often seen using this reagent. In contrast, only a single band was ever seen, corresponding to intergrin-α6, following pull-down and immunoprecipitation experiments, and could be visualised using a less sensitive chemiluminescence reagent (Western Bright, Advansta) as seen in Fig 7A, B of the main text.

Supplementary figure 6

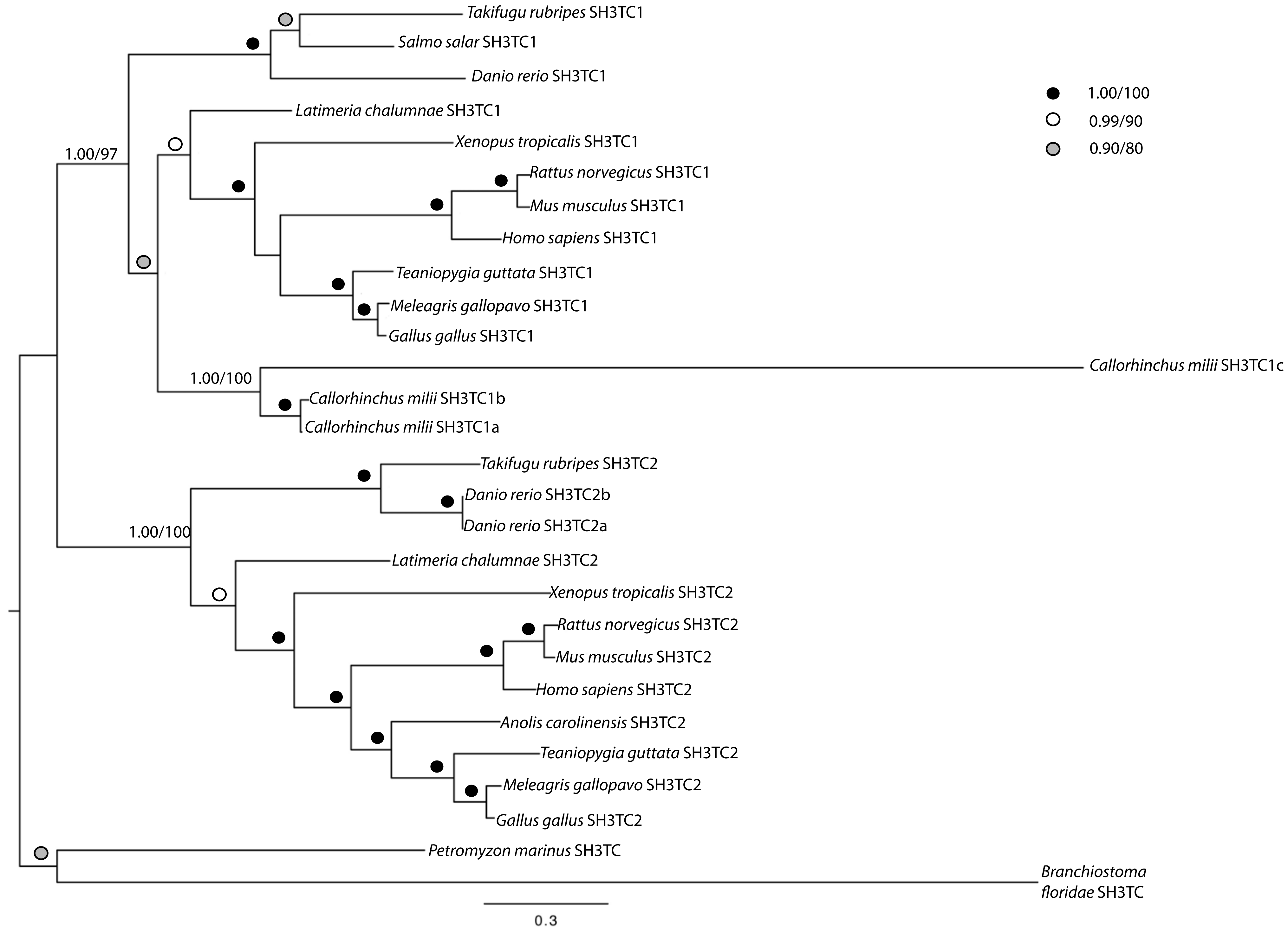

Supplementary figure 6

Phylogenetic analysis of SH3TC homologues, including *C. milii* SH3TC-1c sequence. The support values for the nodes defining clades of SH3TC1 and 2 are robustly supported, even in the presence of the long branch produced by the *C. milii* SH3TC-1c sequence. This strongly suggests that the *C. milii* sequences are legitimate SH3TC1 orthologues and that the duplication giving rise to the SH3TC2 paralogue was concurrent with the evolution of jawed vertebrates. This is the best PhyloBayes topology, with posterior probability values and RAxML bootstrap values shown at key nodes. Other node values are symbolized as inset. The scale bar represents 0.3 changes per site.
